# Age as a modifier of the effects of chemoradiotherapy with infusional 5-fluorouracil after D2 dissection in gastric cancer

**DOI:** 10.18632/aging.203223

**Published:** 2021-07-05

**Authors:** Hung-Chang Wu, Wen-Li Lin, Chien-Liang Lin, Cheng-Yao Lin, Shang-Wen Chen, Yan-Xun Chen, Chao-Hsun Chen, Sung-Wei Lee, Shang-Hung Chen, Chao-Jung Tsao, Wen-Tsung Huang, How-Ran Guo

**Affiliations:** 1 Division of Hematology and Oncology, Department of Internal Medicine, Chi-Mei Medical Center, Tainan, Taiwan; 2 Division of Hematology and Oncology, Department of Internal Medicine, Chi-Mei Medical Center, Liouying, Tainan, Taiwan; 3 Department of Environmental and Occupational Health, National Cheng Kung University, Tainan, Taiwan; 4 Department of Senior Welfare and Services, Southern Taiwan University of Science and Technology, Tainan, Taiwan; 5 Department of Radiation Oncology, Chi-Mei Medical Center, Liouying, Tainan, Taiwan; 6 National Institute of Cancer Research, National Health Research Institutes, Tainan, Taiwan; 7 Department of Oncology, National Cheng Kung University Hospital, College of Medicine, National Cheng Kung University, Tainan, Taiwan; 8 Department of Occupational and Environmental Medicine, National Cheng Kung University Hospital, Tainan, Taiwan

**Keywords:** gastric cancer, D2 dissection, chemoradiotherapy, infusional 5-fluorouracil, elderly

## Abstract

Adjuvant concurrent chemoradiotherapy (CCRT) is the standard care for patients with resected advanced gastric cancer, but its survival benefits remain undetermined in patients undergoing D2 lymph node dissection (D2 dissection). We evaluated safety and efficacy of adjuvant CCRT with 5-fluorouracil (5-FU) versus chemotherapy alone in 110 gastric cancer patients with D2 dissection treated in Taiwan between January 2009 and January 2013. All the 71 patients receiving adjuvant CCRT were treated with daily infusional 5-FU and radiotherapy. Adjuvant CCRT was associated with higher risks of major hematologic (56.3% vs. 23.8%, *p* = 0.002) and gastrointestinal (46.9% vs. 14.3%, *p* = 0.027) toxicities and death (12.5% vs. 0.0%, *p* = 0.041) in patients above 70 years old, but this was not the case in those ≤70 years of age. Univariate Cox proportional regressions identified adjuvant CCRT as a factor for better overall survival (OS) (hazard ratio [HR]=0.52; 95% confidence interval [CI]: 0.27–0.99) and disease-free survival (DFS) (HR=0.46, 95% CI: 0.24–0.88), but it was not a significant factor for OS or DFS after adjusting for other factors in the multivariate analysis. However, in stratified analyses by age, we found adjuvant CCRT was an independent prognostic factor for better OS (HR=0.07; 95% CI: 0.01–0.38) in patients ≤70 years old, but not in those above 70 years of age. Therefore, it was concluded that age may to be a modifier of the effects of adjuvant CCRT.

## INTRODUCTION

Although the worldwide incidence of gastric cancer has declined rapidly over recent decades, the incidence and mortality rates remain high in many geographic regions, including Eastern Asia [1, 2]. The curative treatment of gastric cancer requires gastric resection, but the high mortality rate indicates the prevalence of advanced disease at diagnosis [3]. In a population-based study, after complete resection of gastric cancer, the 5-year survival rate of patients with stage I disease was approximately 70%, while it dropped to 35% or less in stage II disease and beyond [3]. High risks of locoregional recurrence and distant metastasis have also been reported after complete resection in advanced gastric cancer [4]. Therefore, adjuvant treatments, including chemotherapy with or without radiotherapy following surgery, are critical in improving survival in patients with advanced gastric cancer.

The landmark phase III trial, US Intergroup 0116 (INT-0116), has provided promising data in support of adjuvant concurrent chemoradiotherapy (CCRT) following complete resection [5]. The study demonstrated survival benefits associated with CCRT after surgery, and CCRT has thus become standard post-operative care in patients with gastric cancer. Nevertheless, the survival benefits of adjuvant CCRT in patients with D2 lymph node dissection (D2 dissection) remain undetermined because the number of such patients enrolled in that study was limited. Compared to a D0 and D1 lymphadenectomy, D2 dissection is an extensive surgery, entailing removal of lymph nodes along the left gastric artery, common hepatic artery, celiac artery, splenic hilum, and splenic artery. Currently, D2 dissection has been the most widely accepted surgical procedure for treating advanced gastric cancer in Asian and European countries [6–8], but the benefit from adjuvant CCRT remains uncertain in patients with gastric cancer who receive this extensive surgery.

In addition to surgical techniques, the other concern about the INT-0116 is the outdated chemotherapy regimen integrated in CCRT. The chemotherapy regimen of bolus 5-fluorouracil (5-FU) used in the INT-0116 is considered to be remarkably toxic. The safety profiles of several studies in recent decades have demonstrated that continuous infusional 5-FU is generally more favorable than bolus 5-FU [9]. Therefore, we conducted a study to evaluate the efficacy and safety of adjuvant CCRT with infusional 5-FU in patients with gastric cancer after D2 dissection through a comparison with patients receiving chemotherapy alone.

## RESULTS

### Patient characteristics

**Figure 1:**
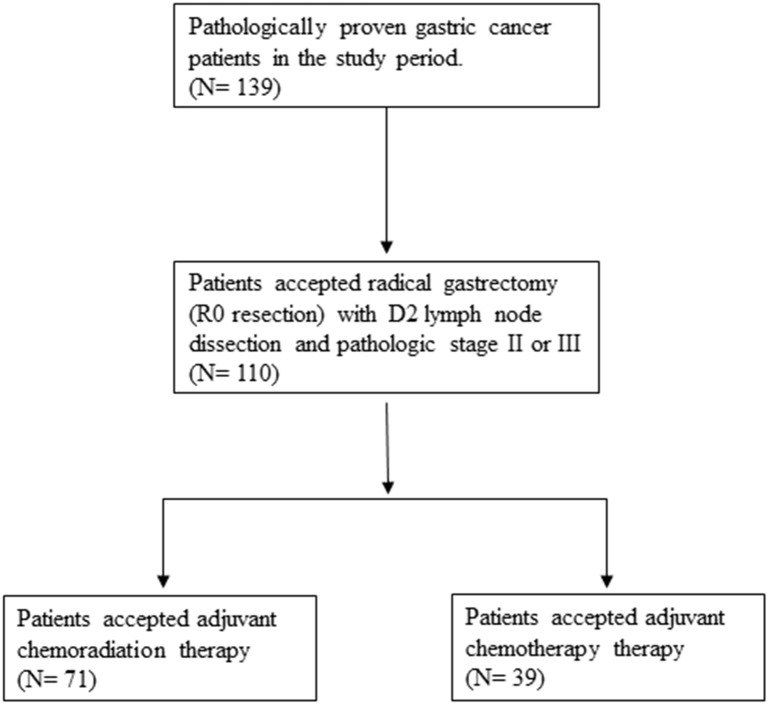
Flowchart of selection of participants.

Between January 2009 and January 2013, a total of 110 patients (66 males and 44 females) who underwent curative resection for gastric cancer with D2 dissection at a medical center in Taiwan were recruited. (Figure 1) The median age was 67 years old, and the range of age was from 37 to 86 years old. Among these patients, 71 received adjuvant CCRT, and 39 received chemotherapy alone. The two groups had similar demographic characteristics, including age and sex, as well as most clinical features, including the Charlson score, ECOG performance status, and pathologic stage [10] (Table 1). Patients in the CCRT group were more likely to have a lower pT stage (1 or 2; 35.2% vs. 15.4%, *p* = 0.024), a higher pN stage (1, 2 or 3; 90.1% vs. 46.2%, p = 0.032), and well or moderate pathological differentiation (25.4% vs. 12.8%, *p* = 0.021).

**Table 1:** Patient characteristics.

**Characteristic^1^**	**All patients** **(n =110)**	**CCRT** **(n =71)**	**CT alone** **(n =39)**	***p* value**
Age (years)				0.067
>70	53 (48.2)	32 (45.1)	21 (53.8)	
≤70	57 (51.8)	39 (54.9)	18 (46.2)	
Sex				0.841
Male	66 (60.0)	42 (59.2)	24 (61.5)	
Female	44 (40.0)	29 (40.8)	15 (38.5)	
Charlson score				> 0.999
≥1	30 (28.2)	20 (28.2)	10 (25.6)	
<1	80 (71.8)	51 (71.8)	29 (74.4)	
Performance status				0.718
0	101 (91.8)	66 (92.9)	35 (89.7)	
1	9 (8.2)	5 (7.1)	4 (10.3)	
Pathologic stage				0.842
II	47 (42.7)	31 (43.7)	16 (41.0)	
III	63 (57.3)	40 (56.3)	23 (59.0)	
pT stage				**0.024**
pT1–2	31 (28.2)	25 (35.2)	6 (15.4)	
pT3–4	79 (71.8)	46 (64.8)	33 (84.6)	
pN stage				**0.032**
pN0	28 (25.5)	7 (9.9)	21 (53.8)	
pN1–3	82 (74.5)	64 (90.1)	18 (46.2)	
Pathological differentiation				**0.021**
Well-Moderate	23 (20.9)	18 (25.4)	5 (12.8)	
Poor	87 (79.1)	53 (74.6)	34 (87.2)	
Overall survival	73 (66.4)	51 (71.8)	22 (56.4)	**0.040**
Disease-free survival	74 (67.3)	52 (73.2)	22 (56.4)	**0.006**

CCRT: concurrent chemoradiotherapy; CT: chemotherapy.

^1^Data presented as n (%).

### Toxicities of adjuvant treatment

All of the patients in the CCRT group completed a course of radiotherapy. The mean dose of 5-FU administrated to each patient was 21293 mg in the CCRT group and 20904 mg in the chemotherapy-alone group (*p* = 0.789). Most common major toxic effects (grade 3 or 4) during adjuvant treatment were hematologic and gastrointestinal toxicities (Table 2). The most common hematologic toxic effect was leukopenia, and severe thrombocytopenia and anemia were uncommon. Common major gastrointestinal toxic effects included nausea, vomiting, and diarrhea. Overall, differences in the incidence of major hematologic and gastrointestinal toxicities between the two groups did not reach statistical significance. Nevertheless, in the patients above 70 years old, patients in the CCRT group were more likely to develop both major hematologic (56.3% vs. 23.8%, *p* = 0.002) and gastrointestinal (46.9% vs. 14.3%, p = 0.027) toxicities than patients in the chemotherapy-alone group. Furthermore, five treatment-related deaths occurred in the CCRT group, including four in patients above 70 years old, but there were none in the chemotherapy-alone group (12.5% vs. 0.0%, *p* = 0.041).

**Table 2:** Major side effects.^1^

	**All patients** **(n =110)**		***p* value^4^**	**Age≤70** **(n=57)**		***p* value^4^**	**Age>70** **(n=53)**		***p* value^4^**
	**CCRT** **(n=71)**	**CT alone** **(n=39)**		**CCRT** **(n=39)**	**CT alone** **(n=18)**		**CCRT** **(n=32)**	**CT alone** **(n=21)**	
Hematologic^2^	25 (35.2)	7 (17.9)	0.079	7 (17.9)	2 (11.1)	0.712	18 (56.3)	5 (23.8%)	0.002
Gastrointestinal^3^	22 (30.1)	9 (23.1)	0.507	7 (17.9)	6 (33.3)	0.538	15 (46.9)	3 (14.3%)	0.027
Death	5 (7.0)	0 (0.0)	0.520	1 (2.6)	0 (0.0)	> 0.999	4 (12.5)	0 (0%)	0.041

CCRT: concurrent chemoradiotherapy; CT: chemotherapy.

^1^Major toxic effects were defined as those of grade 3 or higher; data presented as n (%).

^2^Leukopenia, anemia, or thrombocytopenia.

^3^Nausea, vomiting, or diarrhea.

^4^Chi-squared test or Fisher exact test.

### Survival analyses

With a median follow-up period of 38.3 months (range, 6.0 to 80.0 months), the 3-year overall survival (OS) and disease-free survival (DFS) rates were 83.3% and 72.2% in the CCRT group, and 70.2% and 59.3% in the chemotherapy-alone group. Throughout the study period, the CCRT group had better OS (71.8% vs. 56.4%, *p* = 0.040) and DFS (73.2% vs. 56.4%, *p* = 0.006) than the chemotherapy-alone group (Table 1).

**Figure 2:**
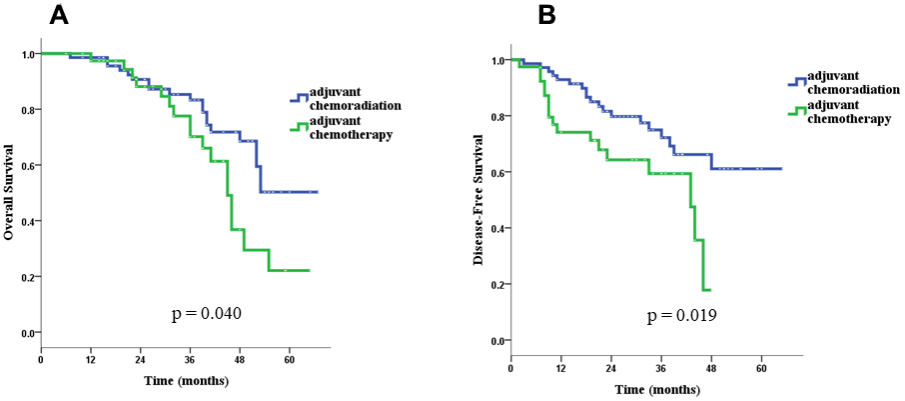
Overall survival (**A**) and disease-free survival (**B**) for the CCRT group and the CT-alone group in the whole group of patients.

**Figure 3:**
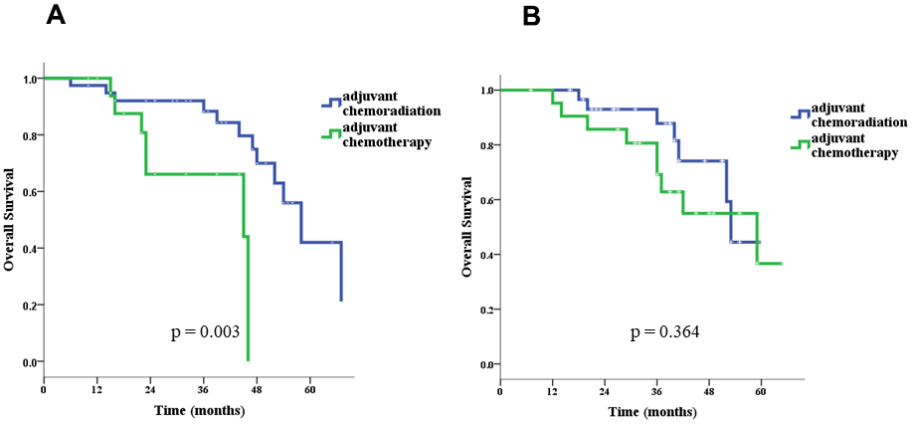
Overall survival in patients ≤ 70 (**A**) and > 70 (**B**) years old.

**Figure 4:**
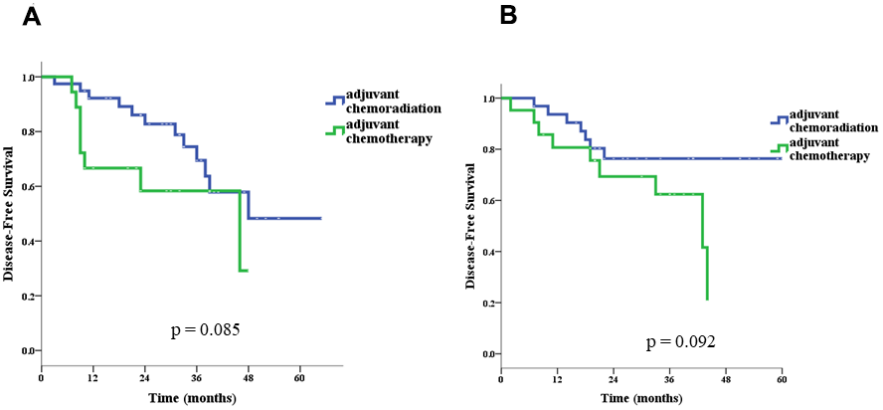
Disease-free survival in patients ≤ 70 (**A**) and > 70 (**B**) years old.

In univariate survival analyses, the CCRT group had both better OS (*p* = 0.040 for log-rank test) and DSF (*p* = 0.019) than the chemotherapy-alone group (Figure 2). Age > 70 years (hazard ratio [HR]= 2.92, 95% confidence interval [CI]: 1.41–6.03), pN stage 0 (HR= 0.40,95% CI: 0.21–0.79), and adjuvant CCRT (HR= 0.53, 95% CI: 0.27–0.99) were identified as significant factors for OS, and Charlson score ≥ 1 (HR= 3.68, 95% CI: 1.06–12.32), pathologic stage II (HR= 0.43, 95% CI: 0.21–0.86), pT stage 1 or 2 (HR= 0.09, 95% CI: 0.04–0.23), pN stage 0 (HR= 0.25, 95% CI: 0.13–0.50), and adjuvant CCRT (HR= 0.46, 95% CI: 0.24–0.88) were identified as significant factors for DFS (Table 3). Because more treatment-related deaths occurred in patients above 70 years old, we performed stratified analyses by age and found that adjuvant CCRT was also associated with better OS (*p* = 0.003) and DFS (*p* = 0.085) in patients younger than 70 years old (Figure 3). However, for patient above 70 years old, the survival benefits associated with adjuvant CCRT did not reach statistical significance for OS (*p* = 0.364) or DFS (*p* = 0.092) (Figure 4).

**Table 3:** Univariate analysis of factors associated with overall survival and disease-free survival.

**Factor**	**Cases**	**Overall survival**			**Disease-free survival**		
		**Events**	**Hazard ratio**	**95% Confidence interval**	**Events**	**Hazard ratio**	**95% Confidence interval**
Age (years)			2.92	1.41–6.03**		1.13	0.63–2.35
>70	53	27			20		
≤70	57	10			16		
Sex			0.64	0.32–1.27		0.70	0.35–1.39
Male	66	25			25		
Female	44	12			11		
Charlson score			1.70	0.41–7.12		3.68	1.06–12.32*
≥1	31	14			9		
<1	79	23			27		
Performance status			0.59	0.23–1.52		0.71	0.25–2.00
0	101	32			34		
1	9	5			2		
Pathologic stage			0.72	0.35–1.49		0.43	0.21–0.86*
II	47	14			16		
III	63	23			20		
pT stage			0.85	0.66–1.10		0.09	0.04–0.23**
pT1–2	31	10			5		
pT3–4	79	27			31		
pN stage			0.40	0.21–0.79**		0.25	0.13–0.50**
pN0	28	16			3		
pN1–3	82	21			32		
Pathological differentiation			0.72	0.35–1.49		0.64	0.31–1.33
Well-Moderate	23	7			9		
Poor differentiation	87	30			27		
Treatment modality			0.52	0.27–0.99*		0.46	0.24–0.88*
CCRT	71	20			19		
Chemotherapy alone	39	17			17		

CCRT: concurrent chemoradiotherapy.

*p < 0.05, **p < 0.01.

In multivariate analyses, we found that age > 70 years (adjusted HR= 2.75, 95% CI: 1.30–5.82) and pN stage 1 or 2 (adjusted HR= 1.93, 95% CI: 1.01–3.69) were independent factors for OS (Table 4). For DFS, pT stage 3 or 4 was an independent factor, with an adjusted HR of 7.43 (95% CI: 2.61–21.40).

**Table 4:** Multivariate analysis of factors associated with overall survival and disease-free survival.

**Endpoint**	**Factor**	**Hazard ratio**	**95% Confidence interval**
Overall survival			
	Age >70 years	2.75	1.30–5.82**
	pN stage 1–3	1.93	1.01–3.69*
	CCRT	0.67	0.34–1.31
Disease-free survival			
	Charlson score ≥1	3.01	0.84–10.76
	Pathologic stage III	1.44	0.68–3.03
	pT stage 3–4	7.47	2.61–21.40**
	pN stage 1–3	1.45	0.66–3.17
	CCRT	0.67	0.34–1.35

CCRT: concurrent chemoradiotherapy.

*p < 0.05, **p < 0.01.

Because the effects of adjuvant CCRT appeared to vary between patients ≤ 70 and > 70 years old in the univariate analyses (Figures 2, 3), we conducted stratified analyses by age according to the results of the multivariate analyses. We found that adjuvant CCRT was associated with better OS in patients ≤ 70 years (adjusted HR= 0.07, 95% CI: 0.01–0.38), but not in patients above 70 years old (adjusted HR= 1.15, 95% CI: 0.53–2.49) (Table 5). In contrast, a higher pN stage (1, 2 or 3) was associated with worse OS in patients above 70 years old, but the effects on patients ≤ 70 years old did not reach statistical significance. For DSF, while adjuvant CCRT appeared to be associated with similar favorable effects on patients both ≤ 70 years old and above 70 years old (adjusted HR= 0.46, 95% CI: 0.19–1.14 for patients ≤ 70 years old; adjusted HR= 0.44, 95% CI: 0.16–1.18 for patients above 70 years old), the HRs did not reach statistical significance (Table 5). A higher pT stage (3 or 4) was associated with similar worse DFS in patients both ≤ 70 years old (adjusted HR= 11.47, 95% CI: 3.51–37.52) and above 70 years old (adjusted HR= 11.36, 95% CI: 2.89–61.75).

**Table 5:** Stratified multivariate analysis of factors associated with overall survival and disease-free survival by age.

**Endpoint**	**Factor**	**Hazard ratio**	**95% Confidence interval**
Overall survival			
>70 years old	pN stage 1–3	3.60	1.56–8.34**
	CCRT	1.15	0.53–2.49
≤70 years old	pN stage 1–3	2.84	0.73–11.02
	CCRT	0.07	0.01–0.38**
Disease-free survival			
>70 years old	pT stage 3–4	13.36	2.89–61.75**
	CCRT	0.44	0.16–1.18
≤70 years old	pT stage 3–4	11.47	3.51–37.52**
	CCRT	0.46	0.19–1.14

CCRT: concurrent chemoradiotherapy.

*p < 0.05, **p < 0.01.

To evaluate the effects of age on the association between adjuvant CCRT and OS, we conducted a further analysis using a multivariate Cox proportional hazards model that included age, adjuvant CCRT, and pN stage, as well as an interaction term of age and adjuvant CCRT. We found a significant interaction between age and adjuvant CCRT on OS, with *p* = 0.008, indicating a modification of the effect of adjuvant CCRT on OS by age. The pN stage appeared to be an independent predictor for OS, and the pN stage 0 was associated with an adjusted HR of 0.28 (95% CI: 0.13–0.58).

### Pattern of relapse

The site of the first relapse was categorized as a local recurrence, peritoneal carcinomatosis, or distant metastasis. Local recurrence was defined as recurrence at the anastomosis site, duodenal stump, tumor bed, remnant stomach, or the regional lymph nodes within the radiation field. Peritoneal carcinomatosis was defined as tumor recurrence at any part of the parietal and visceral peritoneum. Distant metastasis was defined as lymph node recurrence outside the radiation field, liver metastasis, or metastasis in other extra-abdominal sites. We found that patients treated with adjuvant CCRT were less likely to develop peritoneal carcinomatosis compared to those who received chemotherapy alone (5.6% vs. 23.1%, *p* = 0.011), while the incidence rates of local recurrence (8.5% vs. 7.7%, *p* > 0.999) and distant metastasis (12.7% vs. 12.8%, *p* = 0.983) were similar between the two groups.

## DISCUSSION

Benefits of 5-FU-based adjuvant CCRT in gastric cancer were shown in the INT-0116 [5], but whether it also benefits patients after D2 dissection has remained uncertain. A criticism of the INT-0116 was the limited extent of the surgical procedure in most cases, and only 10% of the patients underwent D2 dissection. A retrospective study analyzing several Dutch phase I/II trials [11] found fluoropyrimidine-based adjuvant CCRT to be associated with lower recurrence rates only after D1 lymph node dissection, but not following D2 dissection. Additional evidence of the efficacy of adjuvant CCRT was provided by the ARTIST trial [12], which directly compared adjuvant CCRT to chemotherapy in patients treated with D2 dissection. This Korean study suggested better outcomes with CCRT after D2 dissection only in node-positive gastric cancer patients. In the present study, we compared the efficacy and safety between adjuvant CCRT and chemotherapy alone in patients with gastric cancer receiving D2 dissection. It was found that adjuvant CCRT was associated with better OS and DFS in univariate analyses. In multivariate analyses, after adjusting for other factors, the adjusted HRs associated with adjuvant CCRT increased but did not reach statistical significance. However, in the stratified analyses by age, we found adjuvant CCRT to be an independent prognostic factor for better OS in patients ≤70 years old, but not for patients above 70 years old, indicating a modification effect of age on the benefits.

The survival benefits of adjuvant CCRT after D2 dissection have also been reported in several non-comparative studies [13–16]. The 3-year OS rates of these studies were around 60–70%, superior to the survival rates observed in the INT-0116. This discrepancy in survival rates may have been partially due to the differences in the proportions of patients with D2 dissection in these studies and those in the INT-0116. In our study, the 3-year DFS and OS of the CCRT group were 72.2% and 83.3%, respectively, which were comparable to findings in previous studies on D2 dissection. In addition, we found that patients receiving adjuvant CCRT had better DFS and OS compared to those receiving chemotherapy alone, but the differences (33% reduction in both) did not reach statistical significance after adjusting for other factors.

Although there have been some attempts reported to develop treatment strategies for elderly gastric cancer patients [17], data in the literature regarding the treatment effects of CCRT after D2 dissection on elderly patients with gastric cancer are limited, let alone comparisons between different age groups. In the stratified analyses, we observed very different effects of adjuvant CCRT on OS in different age groups. Namely, significant favorable effects were observed in patients younger < 70 years old, but not in those who were ≥ 70 years old (Table 5). In fact, in patients ≥ 70 years old, the HR associated with adjuvant CCRT was greater than 1, which indicated an unfavorable effect although it was close to 1 and not statistically significant. Our results suggest that the effects of adjuvant CCRT are modified by age, i.e., aging seems to reduce its favorable effects on the OS of patients with gastric cancer after D2 dissection. This appears to be a novel finding.

Gastric cancer may spread to the peritoneum [18–20], and patients with peritoneal carcinomatosis are regarded to be untreatable and have a short life expectancy. Therefore, in the current study, the higher incidence of tumor relapse in the form of peritoneal carcinomatosis may have contributed to the worse OS rates in patients treated with chemotherapy alone (Table 1). Given the dismal prognosis related to peritoneal dissemination, a strategy aimed at the prevention of peritoneal carcinomatosis seems to be a plausible approach to promote survival. Although further direct data from larger prospective studies are warranted, when we excluded patients with peritoneal carcinomatosis and reanalyzed the data from our study, the difference in OS between the two treatment groups did not reach statistical significance, suggesting that adjuvant CCRT may be a feasible treatment to prevent or at least delay tumor relapse in the form of peritoneal carcinomatosis.

5-FU, an antimetabolite drug [21], is the most commonly used antineoplastic agent applied in CCRT to treat various human malignancies, including gastric cancers. The mechanism of the action of 5-FU and its pharmacologic behavior are influenced by its modes of administration, which include continuous infusion and bolus injection [9]. In the INT-0116, the adjuvant CCRT component was given with bolus 5-FU and leucovorin (LV), and high rates of grade 3/4 hematologic (54%) and gastrointestinal (33%) toxicities were observed. In other human malignancies, such as rectal cancer, better tolerability and comparable efficacy of daily low-dose infusional 5-FU have been demonstrated [9]. Consequently, some clinicians utilize continuous infusional 5-FU during adjuvant CCRT rather than bolus 5-FU plus LV in patients with resected gastric cancer. In a pilot study, Leong et al. reported the feasibility of infusional 5-FU-based CCRT, followed by chemotherapy with epirubicin, cisplatin, and 5-FU (ECF), in patients with resected gastric cancer [19]. Although a relatively high rate of grade 3/4 neutropenia was observed (66%), the majority of neutropenic episodes occurred during ECF delivered in the post-radiation period. In another observational study of modified infusional 5-FU-based CCRT [14], Papadimitriou et al. reported that grade 3/4 hematologic toxicities included neutropenia (16%) and thrombocytopenia (3%), while grade 3/4 gastrointestinal toxicities included diarrhea (19%), vomiting (3%), stomatitis/esophagitis (8%), and constipation (3%). These results suggest acceptable tolerability of continuous 5-FU infusion during treatment of CCRT after gastric cancer resection. In our study, the grade 3/4 hematologic and gastrointestinal toxicity rates of adjuvant CCRT were 35.2% and 30.1%, which were comparable with the profiles reported by previous studies on infusional 5-FU-based CCRT [13, 14] and lower than those reported by the INT-0116 using bolus 5-FU during radiotherapy. In addition, the grade 3/4 hematologic and gastrointestinal toxicity rates of patients ≤ 70 years old receiving adjuvant CCRT were both only 17.9% (Table 2), suggesting this therapeutic strategy is a considerably safe treatment option for relatively younger patients with D2 dissection. However, in patients above 70 years old, the grade 3/4 hematologic and gastrointestinal toxicity rates increased significantly to 56.3% (*p* = 0.002) and 46.9% (*p* = 0.027), respectively (Table 2). Most importantly, among these patients, four treatment-related deaths (12.5%, *p* = 0.041) occurred. All these deaths were related to infectious diseases and were observed during the adjuvant chemotherapy period after the completion of CCRT. Although further direct data from larger prospective studies are warranted, when we excluded treatment-related deaths and reanalyzed the data from our study, the difference in OS between the two age groups did not reach statistical significance, suggesting that the high treatment-related mortality contributed to the poor OS observed in patients above 70 years old in comparison with patients ≤ 70 years old in our cohort (Figure 4). Accordingly, the chemotherapy regimen used in this study might not be encouraged in patients above 70 years old with D2 dissection who plan to undergo adjuvant CCRT.

Our study has some limitations. First of all, it was not a randomized trial, and thus was more likely to be affected by confounding factors. However, we evaluated the major potential confounders and adjusted for those that have been identified as likely to affect our results in the multivariate analyses. Secondly, the present study had a relatively small sample size, which limited the statistical power. Nonetheless, it provided enough power to detect some of the differences between patients with and without adjuvant CCRT such as those in the major side effects. It also provided enough power to identify age and pN stage as independent predictors for OS and pT stage as an independent predictor for DSF. More importantly, it even provided enough power to identify the effect modification of age on the benefit of CCRT. The current study was also limited by the follow up period, which was relatively short, not reaching five years in many cases. Therefore, studies with longer follow up periods are needed to confirm the findings.

Still, the current study has some strengths. The chemotherapy regimen did not contain platinum-based antineoplastic agents. Platinum-based agents, including cisplatin, carboplatin, and oxaliplatin, are the most commonly used antineoplastic agents [22]. Some studies have documented the survival benefits of platinum-based agents for patients after D2 dissection. In the multicenter CLASSIC trial, the addition of oxaliplatin after D2 dissection was associated with a significant improvement in 3-year DFS, but grade 3/4 adverse events were also reported in 56% of these patients receiving oxaliplatin [23]. The ARTIST trial randomly assigned patients who had received D2 dissection to adjuvant chemotherapy with capecitabine plus cisplatin (XP) or adjuvant CCRT combined with the administration of XP. It was found that patients receiving XP chemotherapy alone did not have worse 3-year DFS or OS, but grade 3/4 neutropenia occurred in 40.7% of patients receiving cisplatin [12]. To avoid the toxicity accompanying platinum-based agents, the combination of infusional 5-FU-based chemotherapy and integrated CCRT used in the present study is a feasible solution for patients with gastric cancer and D2 dissection who are 70 years old or younger. In addition, using stratified analyses, we observed the modification of the effects of adjuvant CCRT on gastric cancer by age. Specifically, the benefits of adjuvant CCRT on patients with gastric cancer after D2 dissection were limited to those who were 70 years old or younger, and such modification of effects was observed in OS, but not DFS. This further supports the use of the treatment modality on patients ≤ 70 years old.

To evaluate the robustness of the effect modification, we performed two sensitivity analyses: one through stratified analyses by sex, and the other through adding sex to the model. Due to the relatively small sample size, the first analysis can only be done for males, and we found that adjuvant CCRT was associated with an adjusted HR of 1.28 in patients >70 years old while the adjusted HR was only 0.01 in patients ≤70 years old. Although both adjusted HRs did not reach statistical significance, the opposite signs support an effect modification. In the second analysis, the interaction term of age and adjuvant CCRT reached statistical significance (*p* = 0.021), and the regression coefficient was very close to that obtained without sex in the model (-1.78 vs. -2.07). The results also support our argument of an effect modification.

In conclusion, we found that in comparison with chemotherapy alone, infusional 5-FU-based adjuvant CCRT after D2 dissection of gastric cancer had similar safety for patients ≤ 70 years old but was associated with higher risks of hematologic and gastrointestinal side effects and death in patients > 70 years old. In addition, we found the treatment modality was associated with better OS in patients ≤ 70 years old compared to chemotherapy alone, but this was not the case in patients above 70 years old, indicating an effect modification by age. Further prospective studies, especially randomized controlled trials with a larger study sample, are warranted to confirm these results.

## MATERIALS AND METHODS

### Patients

We enrolled patients with gastric cancer who had undergone D2 dissection with removal of at least 15 lymph nodes and fulfilled adjuvant treatment at a medical center in Taiwan between January 2009 and January 2013. Patients were recruited into this study according to the following criteria: 20 years of age or older at diagnosis; Eastern Cooperative Oncology Group (ECOG) performance status of 1 or less; and histologically proven gastric adenocarcinoma, stage II or III, according to the sixth edition TNM staging criteria of the American Joint Commission on Cancer (AICC) [10]. However, patients with any evidence of macroscopic or microscopic residual tumors after surgery were excluded.

### Adjuvant treatment

The time from surgery to the start of the adjuvant regimen ranged between three and six weeks. In patients with adjuvant CCRT, chemotherapy comprised six cycles of a de Gramont regimen (a 2-h infusion of leucovorin 200 mg/m^2^, a bolus injection of 5-FU 400 mg/m^2^, followed by 22-h infusion of 5-FU 600 mg/m^2^ at two week intervals, with the same sequence repeated on the second day). The first two cycles were administered before radiotherapy, and the last four cycles were started after completion of radiotherapy. CCRT consisted of five weeks of radiation with concurrent continuous infusion 5-FU (225 mg/m^2^/day, throughout the entire period of radiation). In the case of the patients treated with chemotherapy alone, eight cycles of the de Gramont regimen were administrated at two week intervals.

The dose of radiotherapy, using an intensity modulated radiotherapy technique, was 45 Gy, with 1.8-Gy daily fractions administered over five weeks. The clinical target volume (CTV) was defined as the tumor/gastric bed, the anastomosis or stumps, and the pertinent nodal groups. Pre-operative diagnostic studies and clip placement were used to identify these regions. The planning target volume (PTV) was created by adding at least one-cm in the superoinferior dimension and radically to the CTV.

### Evaluation of toxicity

Toxicity was assessed using the Common Terminology Criteria for Adverse Events Ver. 3.0 and was evaluated during every treatment cycle. In the event of grade 3 or higher toxicity, chemotherapy was deferred until recovery to grade 1. Dose reduction of chemotherapy was considered if any grade 3 or higher toxicity occurred.

### Follow-up

All patients were regularly monitored after diagnosis until death or their last appointment by the surgeons, medical oncologists, or radiation oncologists at the medical center. We conducted postoperative follow-ups on all patients every three months for two years. After two years, the patients underwent follow-up examinations every six months. At each visit, a physical examination, a complete blood count, and liver function tests were conducted. Chest radiography, abdominal computed tomography, and gastroscopy were performed when clinically indicated. Whenever possible, any suspected recurrence was confirmed by biopsy.

### Statistical analyses

Statistical analyses were performed using Statistical Package for Social Sciences software (SPSS, Ver.17.0, SPSS Inc., Chicago, IL). The major endpoints were OS, defined as the time from the date of diagnosis to the time of last follow-up, or death related to gastric cancer and DFS, calculated from the date of surgery to the date of the first recurrence at any site. Patients lost to follow-up were censored on the latest follow-up date. We evaluated differences in categorical variables using the chi-squared test*,* or the Fisher exact test when it is more appropriate. Because the number of cases was relatively small, we chose 70 years as the cutoff of age, which provided an even division of the study population into two groups and thus provide the maximum statistical power to study the effects of age. Survival analyses were performed using the Kaplan–Meier method with the log-rank test and the Cox proportional hazards regression. To identify independent prognostic factors, we selected factors that were associated with at least marginal statistical significance (*p* < 0.10) in the univariate analyses to conduct the multivariate analyses. All statistical tests were performed at a two-sided significance level of 0.05. This study was reviewed and approved by the Institutional Review Board of Chi-Mei Medical Center (No. 10312-L03).
